# Reporting on the opioid crisis (2000–2018): role of *The Globe and Mail*, a Canadian English-language newspaper in influencing public opinion

**DOI:** 10.1186/s12954-020-00443-7

**Published:** 2020-11-30

**Authors:** Amanda My Linh Quan, Lindsay A. Wilson, Salima S. Mithani, David T. Zhu, A. Brianne Bota, Kumanan Wilson

**Affiliations:** 1 Clinical Epidemiology Program, Ottawa Hospital Research Institute, The Ottawa Hospital, Civic Campus, 1053 Carling Avenue, Box 684, Ottawa, ON K1Y 4E9 Canada; 2 Department of Medicine, University of Ottawa, 451 Smyth Rd, Ottawa, ON K1H 8M5 Canada

**Keywords:** Opioids, Canada, Content analysis

## Abstract

**Background:**

We aim to describe the general characteristics of how the Canadian newspaper *The Globe and Mail* reports on opioid-related news, the opioid crisis and its victims, and explore how Canadians’ perceptions of the opioid crisis could have developed over time from this reporting. *The Globe and Mail* has the highest circulation among Canadian newspapers and is Canada’s newspaper of record.

**Methods:**

Reviewers performed independent, blinded bibliometric searches of all *The Globe and Mail* articles archived in the Canadian Periodicals Index Quarterly spanning an 18-year period (1 January 2000–1 June 2018) related to the keywords “opioids” or “drugs and opioids” and “opiates”. Independently and in duplicate, reviewers manually extracted qualitative data from articles and identified emergent themes. Articles were screened independently by both reviewers based on the inclusion criteria. Conflicts were resolved by discussion and consensus. Social representation theory was used as a framework for describing how the opioid crisis is portrayed in Canada.

**Results:**

Our search yielded 650 relevant opioid articles. The number of articles peaked in 2009, 2012, and in 2016, coinciding with major developments in the epidemic. The language used in this discourse has evolved over the years and has slowly shifted towards less stigmatizing language. Content analysis of the articles revealed common social representations attributing responsibility to *pharmaceutical companies, physicians, and foreign countries.*

**Conclusions:**

*The Globe and Mail’s* coverage of the opioid crisis is focused on basic social representations and attributed responsibility for the crisis to a few collectives. A shift toward coverage of the root causes of the opioid epidemic could positively influence the general public’s perception of the opioid crisis and promote deeper understanding of the issue. Journalists face several obstacles to achieve greater focus and framing of the opioid crisis; a closer working relationship between the media and the research community is needed.

## Introduction

Since the late 1990s, there has been a considerable increase in the use of prescription opioids for the relief of chronic pain [1]. This has resulted in increasing levels of non-medical use of prescription opioids and associated increases in morbidity and mortality due to opioid overdoses [2, 3]. Today, North America is witnessing an ever-increasing rate of opioid misuse contributing to substantial drug-related harms, including dependence and both fatal and non-fatal overdose. In the United States, a 200% increase in opioid overdose deaths was observed between 2001 and 2012 [4]. In 2018, approximately 3.7% of the population 12 years and older had misused opioids in the past [5]. In 2016, opioids were responsible for 66% of total overdoses, resulting in 600,000 hospitalizations [6]. Similarly, in Canada, the rate of apparent opioid-related deaths per 100,000 population increased by 40% between 2016 and 2018, from 8.4 to 11.8 deaths [7] and the rate of hospitalizations due to opioid poisoning increased by 53% between 2007 and 2017 [8]. Canadians are the second-highest per-capita consumers of opioids in the world, after only the United States [2], and opioid misuse has become a public health issue of national concern [9]. This trend is aligned with a growing rate of non-medical prescription opioid use in Canada since 1999 [2]. A 2017 Health Canada survey found that roughly one-third of Canadians who used opioids in 2017 used them illicitly [2].

Historically, media coverage of various epidemics has helped shape the policy agenda [10, 11], while also reflecting ongoing policy discussions, debates and developments. As in the case of HIV/AIDS, the news media serves as an important source of information on a public health issue—the opioid epidemic—and contributes to shaping public understanding of the issue [12, 13]. However, evidence from previous studies examining the effects of repetitive news framing, language used and agenda-setting highlight how public perception of a problem’s importance [13, 14] and its potential solutions can be influenced by media coverage. U.S. news media portrayal of the opioid crisis has been shown to influence public perception of the opioid crisis by framing the issue as one of criminal justice rather than of public health. In a 15-year study of the four highest-circulation national U.S. newspapers, over 60% of opioid-related news stories focused on prosecuting drug dealers and enforcing drug laws, relative to only 4% that focused on substance use treatment and/or harm reduction policies [15]. Canadian news media coverage of opioid-related harm reduction strategies showed that Canadian print newspapers are capable of shaping public discourse on developments in harm reduction by selectively reporting on supervised opioid consumption and naloxone, leading to increased public support for these strategies compared to other evidenced-based alternatives (e.g. safer inhalation) [16]. However, a comprehensive portrayal of the opioid crisis that is not limited only to harm reduction is still poorly understood, especially with regards to how it evolved over the previous two decades [17]. Thus, to better understand how opioid-related media coverage could have impacted the perceptions of Canadians, we analysed the portrayal of the opioid crisis in Canada’s English-language newspaper of record, *The Globe and Mail*.

## Theoretical framework

Social representation theory (SRT) [18] focuses on explaining how social representations of public issues are formed and how these social representations help individuals, groups and communities make sense of unfamiliar, problematic events or issues. SRT has been used as a framework for investigating complex and challenging social phenomena such as HIV/AIDS [19, 20], climate change [21, 22], and poverty/homelessness [23]. Anchoring, one of the processes by which social representations are formed, involves integrating unfamiliar objects into pre-existing knowledge and fitting novel information into previously formed ideas, thus making unfamiliar concepts familiar [24, 25]. This is often done by attributing responsibility to specific collectives (e.g. large institutionalized groups like pharmaceutical corporations, foreign nations, professions, etc.). Determining how the media could have impacted social representation of the opioid crisis by attributing responsibility to different collectives over time provides a deeper understanding of how the issue could be understood and perceived by every-day Canadians.

This study sought to: (1) describe general characteristics of how *The Globe and Mail* reports on opioid-related news, the opioid crisis and its victims and (2) explore how Canadians’ perceptions of the opioid crisis could have been impacted by a prominent source of Canadian news media using the framework of social representation theory.

## Methods

### Data collection

Newspaper reporting remains an important source of information for Canadians; as of February 2018, 58% Canadians aged 18 or above read any kind of print newspaper at least once a week [17]. In order to capture the magnitude and nature of newspaper coverage of the opioid crisis, we conducted a thematic analysis of articles in Canada’s national paper of record—*The Globe and Mail. The Globe and Mail* had the widest circulation and highest print readership in 2018 (6.5 million print and digital readers) [26, 27]*. The Globe and Mail* prints separate editions for the following cities across Canada: Montreal, Toronto, Winnipeg, Calgary and Vancouver, featuring a combination of national stories and local stories. Their target readership base consists of individuals with a household income of $120,000, a post-secondary education, and who are commonly in positions of greater policy- and decision-making influence [28]. Historically, *The Globe and Mail* has played key roles in agenda-setting for a wide array of issues [29] making it an ideal source to analyze for the purpose of elucidating how a leading and influential source of Canadian news media could have impacted Canadians’ public perception of the opioid crisis and the surrounding discourse. *The Globe and Mail* also does not have a strong political slant like the next two largest circulation dailies (National Post and Toronto Star).

Two reviewers independently performed a bibliometric search of all *The Globe and Mail* articles published and archived in the Canadian Periodicals Index Quarterly (CPI.Q), within an 18-year timeframe (1 January 2000–1 June 2018). Three search terms that were used were: (1) “Opioids”, (2) “Drugs and Opioids”, and (3) “Opiates”. Inclusion criteria were defined as any articles referring to opioid-related news or the epidemic, its victims, and mitigation or harm reduction strategies. Exclusion criteria were defined as any articles focusing on drugs that are neither opioids, opiate derivatives, nor related substances (e.g. fentanyl, heroin, morphine), or that focused on subjects other than opioids as the main topic. Both reviewers screened articles independently based on the inclusion criteria, then resolved all conflicts by discussion and consensus.

### Data preparation and analysis

A data extraction form was developed as a means of objectively and systematically recording data obtained from the articles. Using this form, reviewers manually extracted qualitative data from articles independently and in duplicate. Information such as date, location, authors, title, drugs mentioned, experts mentioned, language describing people who use opioids, role of opioids (i.e. benefit, harm, or mixed), and potential sources of the issue were extracted to help determine patterns in language, risk portrayals, and shifts in coverage emphasis. In line with the aims of the study and the SRT framework, the articles were also sorted and coded into groups by type of article, type of evidence, location of article and attribution of responsibility. The coding process was performed manually. Due to the evolving nature of the opioid epidemic and the surrounding discourse, the extraction form and coding framework were modified as needed through a joint and iterative process [30]. All sorting and reporting disagreements were resolved by consensus.

## Results

### Overview

**Fig. 1:**
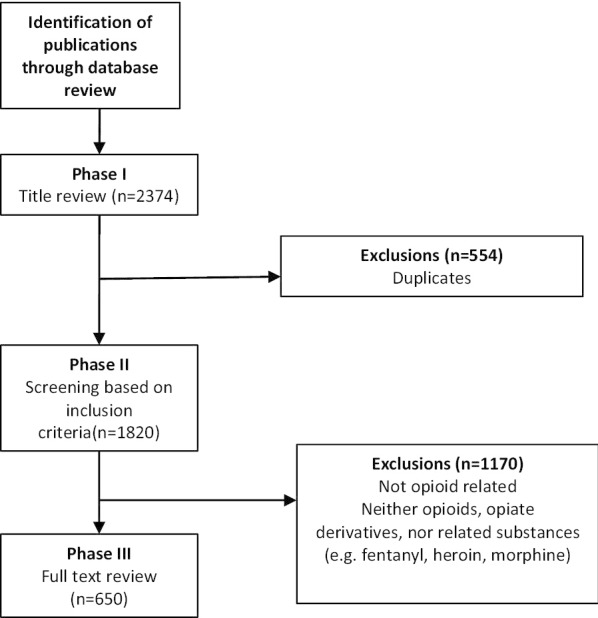
Flow chart for screening

**Fig. 2:**
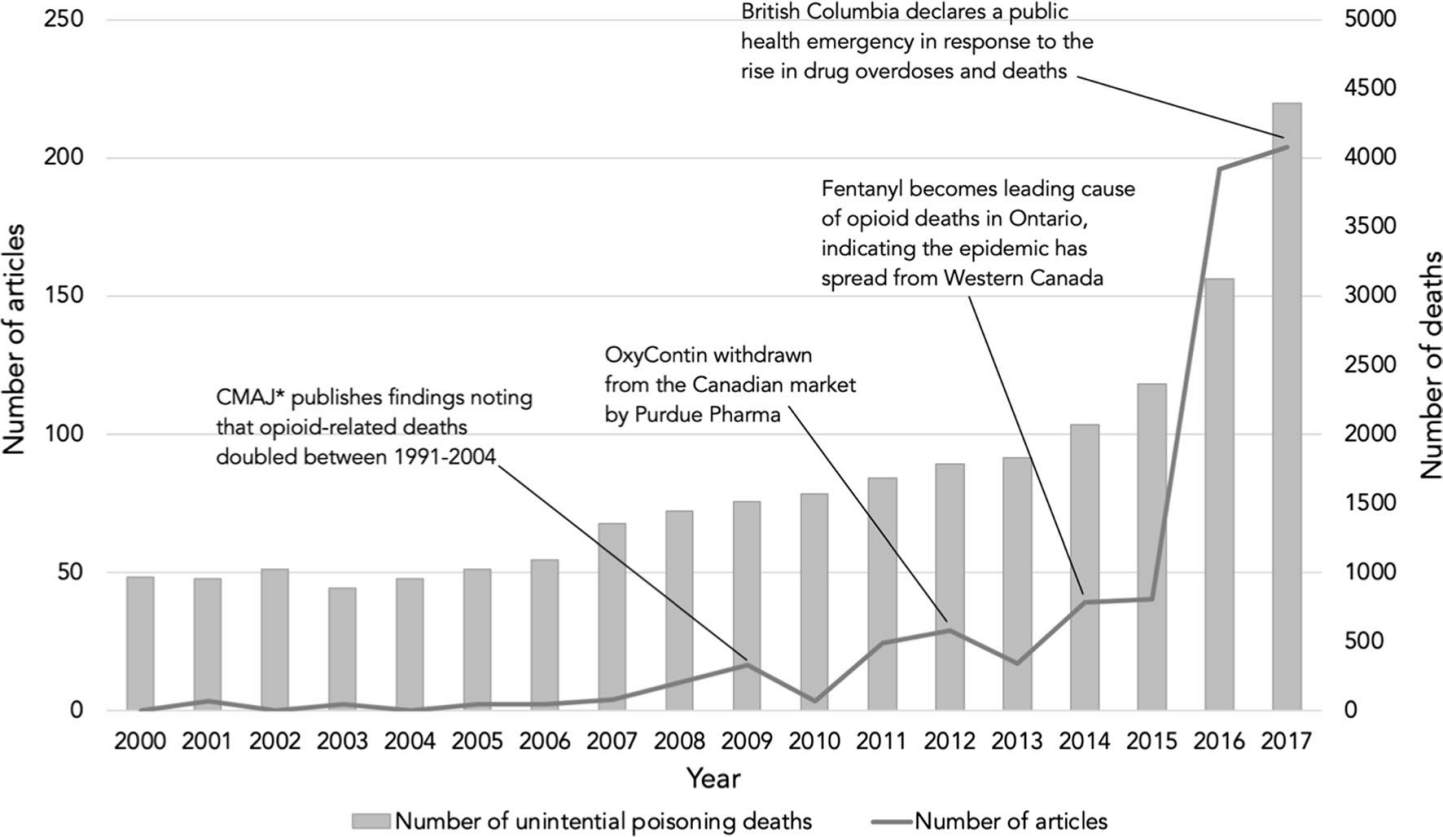
Number of relevant articles relating to search criteria “opioids”, “opioids and drugs”, and “opiates” by *The Globe and Mail,* and annual number of unintentional poisoning deaths in Canada (2007–2017). *CMAJ is the Canadian Medical Association Journal

Our initial search yielded 2374 Globe and Mail articles. Of these, 554 duplicate articles were removed, and the remaining 1820 articles were screened based on the inclusion criteria. We ultimately identified 650 relevant articles for content analysis (Fig. 1). Initial reporting began in 2001; the early years had very few to no opioid-related articles (Fig. 2). The predominant article type was news reports (72%, *n* = 469), although opinion-editorial articles and human-interest stories were popular as well (Table 1). News reports and editorials were typically centered around statements of risk or criminal activity, while the anecdotes and opinion-based stories tended to focus on the far-reaching implications of opioid dependence from either the patient or the healthcare provider perspective. Of the 171 different authors present in this study, the leading authors were Woo, Howlett, Weeks, Giovannetti, and Paperny, with 126, 67, 28, 22, and 18 published articles, respectively.

**Table 1:** Characteristics of articles

Location of article	# of articles (% of total^a^)	Article type	# of articles (% of total)
Banner headline	80 (12.3%)	Human interest stories	50 (7.8%)
Front page other section	137 (21.1%)	News report	469 (72.1%)
Front section (but NOT front page)	322 (49.5%)	Opinion/editorial	121 (18.6%)
Not front page or front section	111 (17.1%)	Other (statistics, exposés etc.)	10 (1.5%)

^a^Note that some articles did not have a location/page listed

The number of opioid-related articles peaked in 2009, again in 2012, and substantially increased in 2016. Manual review of the opioid-related stories revealed that peaks in news coverage often coincided with major developments in or reports on the epidemic (Fig. 2).

## Language and types of evidence

### Main types of evidence

**Fig. 3:**
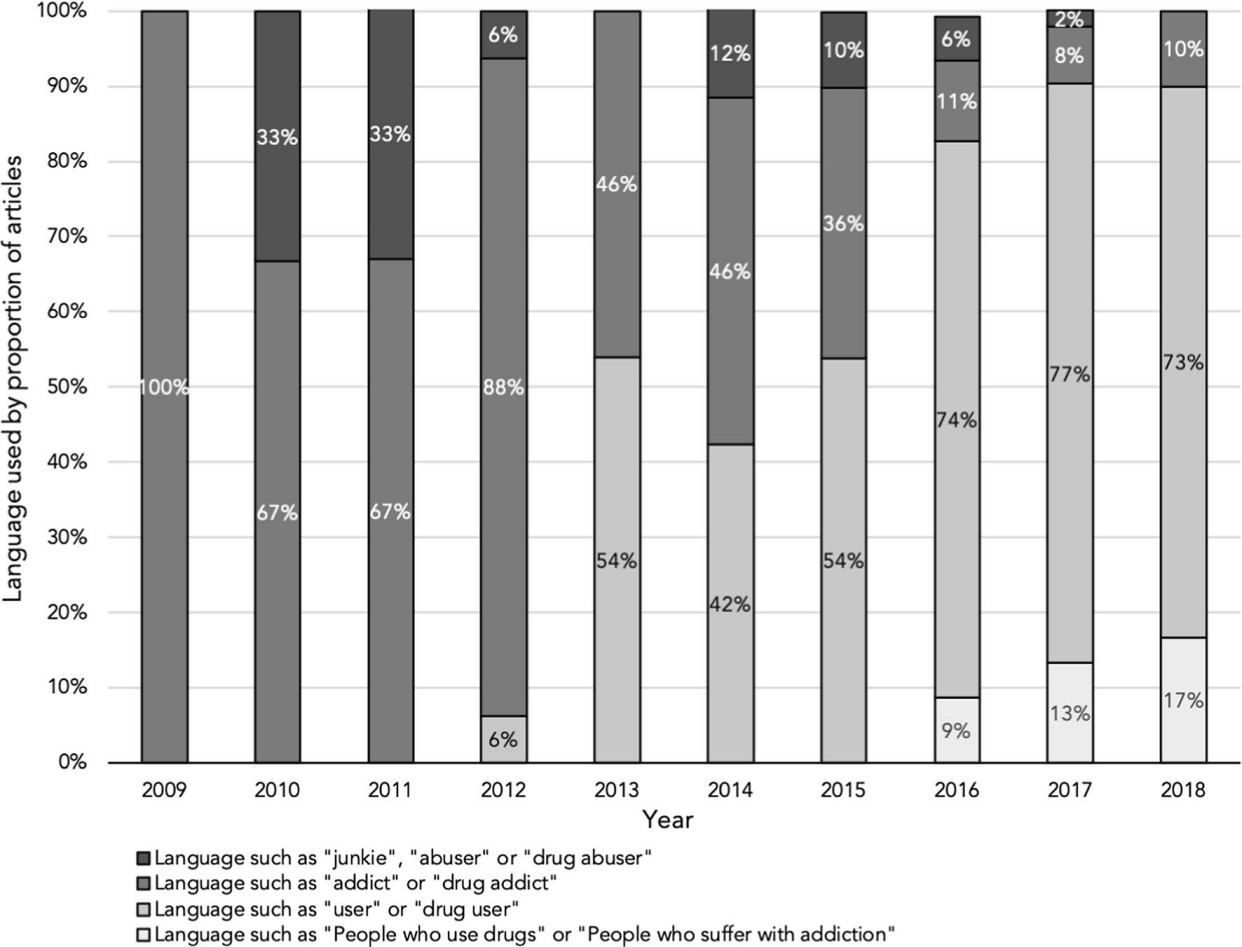
Proportion of articles and language used to describe individuals suffering with substance use disorder (2009–2018)

Of all 650 articles, 22.3% (*n* = 145) referred to general statements of risk as evidence to support their claims and 15.2% (*n* = 99) of articles referred to some form of scientific study as evidence. Many of these statement of risk articles focused on the potency of fentanyl and other illicit drugs that may be mixed with opioids when sold illegally. Articles covering this topic also frequently called for more public awareness, more provincial and federal action to expedite harm reduction programs such as methadone clinics and naloxone training programs and warned about the risks of overdose. For example, the news article “Ontario, Ottawa expand free access to antidote for opioid overdoses” [31] emphasized the prevalence of opioid overdoses in several hotspots (i.e. Ontario, Alberta, and BC), outlined Health Canada’s existing efforts to mitigate opioid overdose deaths (e.g. naloxone kits) and concluded with a demand for better accessibility to and affordability of naloxone kits. Statement of risk articles (56%, *n* = 81) often addressed stigma, the tendency of authority figures to refer to people suffering from addictions as criminals rather than patients, and barriers to effective and timely access to treatment and care (Fig. 3).

Of the articles that referred to some form of scientific study as evidence, the majority occurred between 2008 and 2018 (13.8% *n* = 90). These articles focused on the over-prescription of opioids by physicians and clinical trials for alternatives to over-prescribing such as the NAOMI and SALOME trials which involve the supervised prescription of medical heroin and hydromorphone for patients with more “severe” opioid dependence [32–36].

### Changes in language use over time

From 2009 to 2018, the use of stigmatizing language such as “junkies” and “abuser” in reviewed newspaper articles declined, as did the term “drug addict” (Fig. 3). In 2012, we noted the emergence of the terms “user” and “drug user”, and 2016 onwards, the use of less stigmatizing terminology such as “people who use drugs” or “people who suffer with addiction”.

Interestingly, shifts in the language used over time differed between many authors. Woo tended to focus on covering legal implications of harm reduction actions and overdose prevention, shifting to less stigmatizing language use later (2015–2018) than most other authors. Howlett focused on opioid over-prescription by physicians and consistently used less stigmatizing language like “people addicted to opioids”. Weeks, Giovannetti, and Paperny all frequently covered news on overprescribing and harm reduction, and often referred to the opioid crisis as a “public health issue” rather than a criminal issue. Overall, the shift towards less stigmatizing use of language was closely interlinked with the framing of the opioid crisis as a public health, rather than criminal, issue for all authors.

### Changes in narrative framing over time

A common framing characteristic of media reporting of opioid use and dependence is the use of hyperbole that dramatizes or sensationalizes the issue [37]. Examples include newspaper headlines such as “Drugs ravage picture-perfect community” [38] and “We have opened Pandora's box—it's going to haunt us” [39]. Descriptions such as “teenage girls would do housework in their underwear in return for pills”, “abusers cut up the patches [fentanyl patches] and eat the pieces”[40] and “thefts and break-and-enters so they [people who use drugs] can feed their habits” [41], also demonstrate how the issue was being framed in an inflated manner. This may lead to the formation of harmful stereotypes towards people who use opioids.

From 2001 to 2008, framing of the opioid crisis centred on the abuse of prescription opioids, opioid-seeking behaviour leading to crime and theft, and double-doctoring (seeking prescriptions from multiple physicians) [42, 43]. Further, these articles commonly portrayed society’s dominant attitude towards those who use opioids as “distrustful”, “crime-breeding”, and a “threat to neighbourhoods’ tranquility, public safety and property values”. Notably, methadone programs and supervised injection sites were portrayed as mediums to promote opioid abuse [44]. From 2008–2011, a transition in the framing of the opioid crisis from a criminal and legal issue towards one of public health started to emerge more prominently. For example, articles mentioned that “focusing on policies that criminalize opioid users do not appear to stem appetites for drugs and can further exacerbate issues by the way of stigmatization, mass and disproportionate incarceration, the spread of infectious diseases like HIV and hepatitis, and impeded access to life-saving medications and treatment” [45]. Further support for harm reduction and addiction treatment programs was observed during this period as well, along with more evidence- and research-based support for these programs, like “supervised injection sites have led to a 35% reduction in fatal overdoses within 500 m of the sites, 100% reduction in fatal overdoses in-site, and no increase in local crime rates or public safety concerns” [46]. From 2011 onwards, the opioid crisis continued to be portrayed most often as a public health issue [47].

## Social representation themes and attributed responsibility patterns

**Fig. 4:**
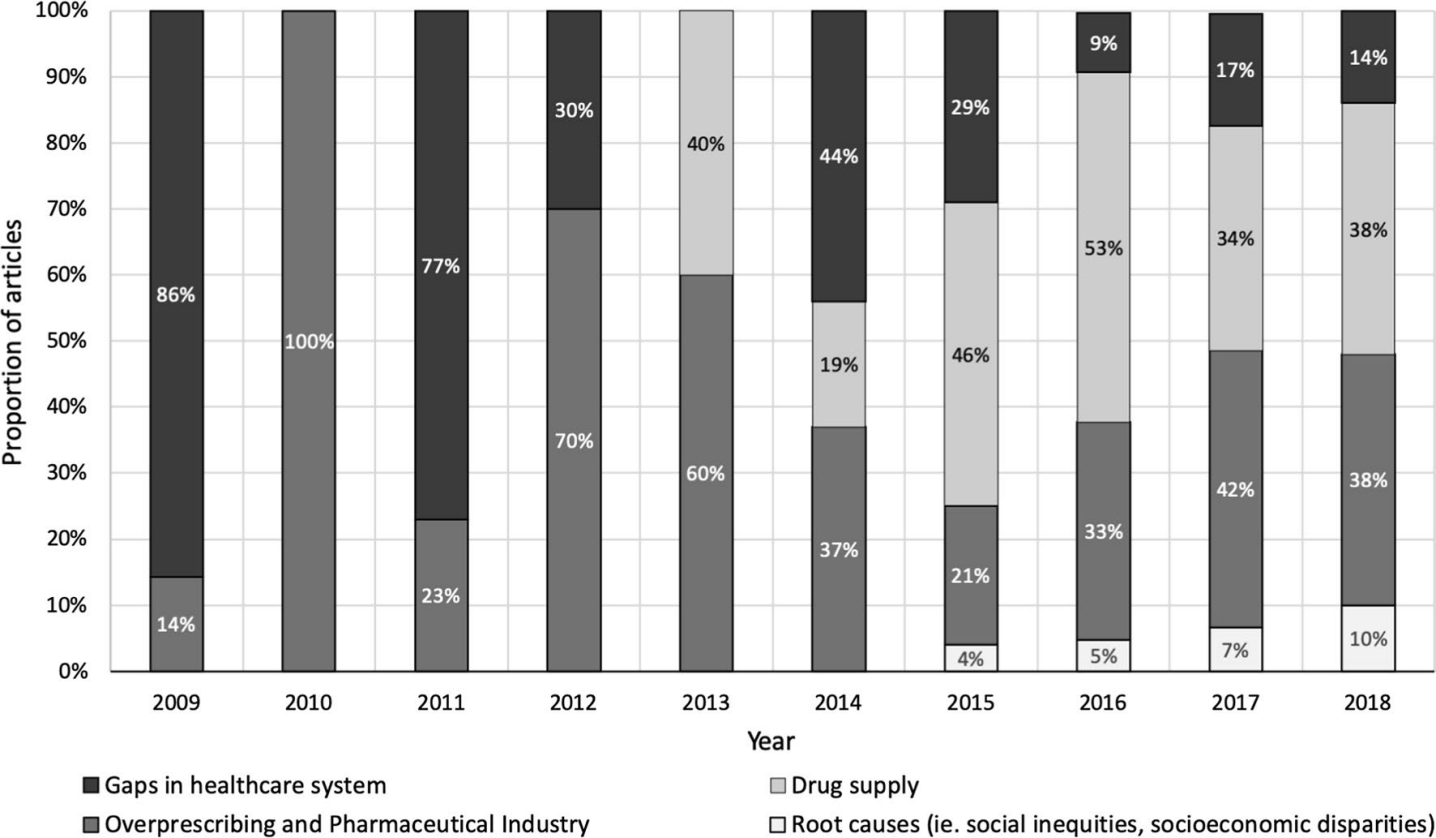
Proportion of articles stratified by response attribution designated (2009–2018). Note Between 2002 and 2008, 14 articles (*n* = 14) were identified for gaps in healthcare which have been excluded from the figure

Content analysis of the articles revealed themes associated with key groups. Some of the frequent social representations among the articles was the attribution of responsibility to pharmaceutical companies or physician overprescribing (*n* = 102, 15.7%), drug supply (*n* = 103, 15.8%), gaps in the healthcare system (n = 60, 9.2%) and root causes of the opioid crisis (*n* = 12, 1.8%). The representation of these in *The Globe and Mail* over time is presented in Fig. 4. Of the 650 total articles, 46% (*n* = 300) mentioned one or more of these social representations as a key factor in contributing to the opioid crisis [48].

The role of the pharmaceutical industry in motivating the massive rise in opioid prescriptions [49, 50], and how, if at all, they were being held responsible for their actions in Canada and abroad were widely discussed. These articles typically claimed that “Big Pharma” placed “profits over patients” by promoting the benefits of opioids to physicians without complete disclosure of the risks. These representations of *pharmaceutical companies* can be easily anchored to the public’s predisposed lack of trust towards the global pharmaceutical industry in prioritizing patients’ health outcomes first [51].

As stewards of the legal opioid supply, *physicians’* role in the creation of the opioid crisis, either by overprescribing, indiscriminately prescribing, or through a lack of proper training and education on pain management leading to liberal prescribing etc., was also discussed. Articles explored out-dated provincial opioid prescribing guidelines [52, 53], and the creation of national opioid prescribing guidelines to improve and standardize prescribing practices [54–56]. The media’s representation of physicians often included reference to influence from the pharmaceutical industry—“doctors began prescribing opioids two decades ago to relieve moderate to-severe pain as pharmaceutical firms promoted their benefits” [57]. Overall, the role of the pharmaceutical industry and/or physicians in the opioid crisis was mentioned in 24% (*n* = 154) of the total articles [48].

Within the overall discourse surrounding the illicit drug supply, responsibility was also frequently attributed to a foreign country. The smuggling of contaminated drugs, often laced with fentanyl, from China into Canada was widely reported: “…it's easy to buy the stuff [fentanyl] from black-market labs in China, with massive quantities of drugs of unknown provenance and quality shipped here under the noses of police and the Canada Border Services Agency” [58]. Articles that addressed foreign countries represented 16% (*n* = 102) of the total articles [59]. An investigation into the source of illicit fentanyl, conducted by *The Globe and Mail* in 2016 [60] was often referenced to confirm that tainted drugs were responsible for an increase in opioid-related deaths. Many articles thereafter detailed drug trafficking [61, 62], and joint efforts between China and Canada to curb such activities [63]. The social representation of China and their role in the opioid crisis can be anchored to the public’s pre-existing concern towards Chinese government activities due to several drug and food safety issues in the past [64–66].

Interestingly, patterns of responsibility attribution in *The Globe and Mail* varied geographically across Canada. National stories, accounting for 14.9% (*n* = 97) of reviewed articles, referred more often to physicians and gaps in the healthcare system than Big Pharma and foreign countries. Similarly, local stories also attributed responsibility predominantly to physicians and gaps in the healthcare system but varied in attributing responsibility to Big Pharma and foreign countries. Local stories in British Columbia focused more commonly on the illicit drug trade from foreign countries than physician prescribing of opioids, whereas local stories from Ontario showed the opposite trend. Examples of B.C. local articles include "RCMP target fentanyl shipments from China" [61] and “China refutes claims it's a major source of fentanyl” [67], which encourage more public awareness of the “contaminated opioids which has led to surges in fatal overdoses in B.C”. Ontario physicians bore the principal responsibility for the creation of national opioid prescribing guidelines [68, 69].

It is important to note that these media representations of responsibility attribution evolved as the long-term consequences of opioid overprescribing became more apparent. Many early articles (2000–2006) focused on the need for opioids for pain management and discussed opioids’ apparent addictiveness [70, 71]. Following this (2007–2012), the vast majority of articles addressed the steadily increasing rates of prescription painkiller use and fatal and non-fatal opioid overdoses [72, 73]. During this time, gaps in the healthcare system (e.g. lack of chronic pain management, access to counselling, harm reduction services, etc.) and overprescribing (also known as liberal prescribing) by physicians with influence from the pharmaceutical industry were held responsible (Fig. 4) [50, 74]. Concurrently, policy-related articles also initially focused on the need for opioid prescribing guidelines for healthcare providers and pharmaceutical tracking systems [75]. From 2012 on, the growing prevalence of substance misuse was thrust into the spotlight with several high-profile overdoses [76, 77] and the delisting of high-dose prescription opioids from province's drug plans [78, 79]. It was also at this time that responsibility attribution patterns shifted away from physicians and the pharmaceutical industry towards a tainted and increasingly potent illicit opioids supply [61, 80]. Next, between 2014 and mid-2016, opioid-related overdoses continued to increase rapidly, and policies focused on better overdose surveillance and expanded access to Naloxone, a drug that can reverse opioid overdose. Throughout the last few years (mid-2016 to 2018), policies focused on approving and opening additional supervised consumption sites [81–83] with the responsibility for opioid-related harms still primarily placed on the illicit drug supply of opioids from China [63, 84].

Only few articles (1.8%, *n* = 12) mentioned the root causes of high-risk drug use, such as social inequity, socioeconomic disparities, pain, and unresolved trauma [85, 86]. Despite the fact that mental health issues are extremely common among people who use drugs, [87], these issues were rarely addressed [88].

## Discussion

Our study offers insight into news coverage of the opioid crisis in Canada from *The Globe and Mail* and highlights its important role in framing issues and acting as a disseminator of information. Of note, while earlier research promoted the use of opioids for pain management [70, 89], this could have contributed to the initial perception of their safety. This is analogous to early academic papers that also minimized risk of opioid dependence or harm and have been extensively cited as evidence of the perceived lack of risk around opioid prescribing [90]. This form of early information can create an anchoring bias that can be difficult to change, even in the presence of evidence to the contrary [91]. In this case, there was a delay between academic evidence suggesting the potential harms associated with opioid use (evidence that appeared in late 1990s to early 2000s) [92] and changes in expert opinion and media reporting, which did not consistently warn about opioid misuse and dependence until 2009. The media’s role in communicating public health information at early stages, as in the case of the opioid crisis, merits further exploration.

We found a significant increase in the coverage of opioids and opioid misuse from 2010 onwards (Fig. 2). Peaks in coverage coincided with major public health or policy developments, indicating that opioids and their misuse became and have remained a priority issue in the eyes of the public over the years (Fig. 2). As suggested by the results of this study, the language used to describe people who use drugs has shifted to less stigmatizing language in recent years. However, the continuing news framing and sensationalizing of opioid issue mimics fear-based opioid campaigns [93] and has the potential to increase the stigma surrounding drug use, shame individuals who use drugs and discourage them from seeking help [94]. While some may argue that this coverage encourages action and staves off complacency, others have stated that fear-based opioid campaigns often fail to motivate people to change their behaviour, may appeal to individuals’ desire for risk-taking or may even foster an attitude of apathy towards substance use-related harms [95]. Drawing parallels to the HIV/AIDS epidemic, a 2005 meta-analysis of 354 HIV-prevention intervention strategies [93] tested the major theoretical assumptions about behaviour change and concluded that the most effective interventions were those that contained attitudinal arguments, educational information, behavioural skills arguments/training, while the least effective strategies were those that attempted to induce fear. Mass media campaigns that portray emotional messages such as personal testimonials, stories, and intense images have also been found to be an effective strategy in smoking cessation and changing smoking behaviours in adults [96, 97]. This could be a beneficial strategy to educate about opioid misuse and change perceptions about the opioid crisis in Canada.

To understand how Canadians’ perception of the opioid crisis could be impacted, we used the SRT framework. This analysis revealed how newspaper coverage could have aided the development of responsibility attribution patterns towards different collectives. It is evident that the current opioid crisis is, and is communicated as, several overlapping crises—over-prescription of pain medications (by pharmaceutical companies and physicians), the growing availability of contaminated street drugs, and a failure to fully implement harm reduction measures (Fig. 4). It is easy for both the media and the public to blame these collectives (i.e. the pharmaceutical industry, physicians, and foreign countries), because the content of the news media contributes to social representations that can be anchored in the public’s predisposed notions. Only 11.4% (*n* = 74) of all articles discussed secondary causes of the opioid crisis, such as gaps in the healthcare system (e.g. lack of funding for or availability of, opioid treatment programs, addiction services, alternative pain management services) [98–100]. Even fewer articles (1.8%, *n* = 12) mentioned the root causes of high-risk drug use—social inequity, socioeconomic disparities and unresolved trauma [85, 86]. Journalists face several obstacles to achieving this more nuanced focus and framing of the opioid crisis, and a closer working relationship between the media and the research community may help to overcome these challenges.

Prior to this study, several other studies have examined media coverage of the opioid crisis [15, 16] but none, to the best of our knowledge, have investigated the issue with the framework of SRT. This study provides novel details about how Canadians’ public perceptions of the opioid crisis may have been shaped by a high-circulation, highly influential English newspaper. This study spans an 18-year period ( 1 January 2000–1 June 2018) and provides a comprehensive overview of how news reporting and focus changed over time.

### Limitations

A major limitation of this work is our inclusion of only one newspaper’s print articles. While it serves as the paper of record, only a subset of the Canadian population reads printed newspapers, and a further subset of those individuals choose to read *The Globe and Mail.* Further, *The Globe and Mail’s* target demographic of highly educated, higher-income people may have resulted in a lack of balanced representation for evaluating public perception [28, 101]. Furthermore, the political leanings and beliefs of *The Globe and Mail* contributors could potentially bias the language and slant of news articles focused on government policies and decisions [28, 101]. In addition, the way in which news is consumed has also changed drastically over the reporting period—today, the large majority of newspaper readers are now accessing at least some of their news online. Roughly 77% of individuals between the ages of 18 and 34 use their phone to access digital news [102]. Another limitation to our work is that we did not include any newspaper articles published in French. Including other news sources and databases may have revealed additional themes that were not included in our analysis. Future studies seeking to understand reporting and in what ways it could influence perceptions of the opioid crisis should take more forms of reporting (e.g. Internet, social media, magazines etc.), content, and languages into consideration.

## Conclusions

The opioid crisis is a complex health and social issue, and journalists play an important role in knowledge dissemination and perception development. Based on our results, we believe that improvements have been made over time to the language used in reference to people who use drugs, but a greater focus by the media on the primary and secondary causes of the opioid crisis is needed. We believe a shift in coverage away from basic social representations and responsibility attribution patterns towards root causes of the opioid epidemic has the potential to positively influence the general public’s perception of the opioid crisis and help reap a deeper understanding of the issue.

## Data Availability

The datasets used and/or analysed during the current study are available from the corresponding author on reasonable request.

## Abbreviation

SRT: Social representation theory
